# Cohort Profile: The Haematological Malignancy Research Network (HMRN): a UK population-based patient cohort

**DOI:** 10.1093/ije/dyy044

**Published:** 2018-04-02

**Authors:** Alexandra Smith, Debra Howell, Simon Crouch, Dan Painter, John Blase, Han-I Wang, Ann Hewison, Timothy Bagguley, Simon Appleton, Sally Kinsey, Cathy Burton, Russell Patmore, Eve Roman

**Affiliations:** 1 Department of Health Sciences, University of York, York, UK; 2 Paediatric Haematology and Oncology Unit, Leeds General Infirmary; 3 St James’s Institute of Oncology, Leeds Teaching Hospitals NHS Trust, Leeds, UK; 4 Queens Centre for Oncology, Castle Hill Hospital, Cottingham, UK

## Why was the cohort set up?

With diverse aetiologies, treatment pathways and outcomes, haematological malignancies comprise a heterogeneous group of over 60 cancers.^1^^,^^2^ Critically for epidemiology, appreciation of the similarities and differences within this complex cancer group only emerged in recent decades, as understanding about the relationship between the various haematological malignancies, the bone marrow, the immune system and the cellular and genetic basis of malignant transformation gradually increased. Integrating genetic data, with information on morphology, immunology and clinical parameters, the first World Health Organization (WHO) consensus classification of haematological malignancies, which is incorporated into the International Classification of Diseases for Oncology (ICD-O3), was published in 2001.^3^ Since then, haemato-oncology has continued to be one of the most rapidly evolving fields in cancer research, with advances in genomics and diagnostic technologies leading to further WHO revisions.^1^^,^^2^^,^^4–6^ Unfortunately, however, although these classification changes have been rapidly adopted into clinical practice, the radical nature of the shift has posed significant problems for population-based cancer registries, with many struggling to capture data on new entities and continuing to report using the traditional ICD-10 groupings of leukaemia, Hodgkin lymphoma, non-Hodgkin lymphoma and myeloma.^7–11^

Population-based data are required not only to inform aetiological hypotheses and plan health care services, but also to monitor the impact of therapeutic changes in the general patient population. This need is particularly pertinent in fast-moving areas like haemato-oncology where treatment protocols are subject to rapid change, and ‘gold-standard’ randomized controlled trials (RCTs) are frequently restricted to specific patient sub-groups: often younger patients with fewer comorbidities.^12–18^ Furthermore, in some countries, particularly those where universal health care is lacking, the likelihood of trial entry often varies with socioeconomic status, gender and ethnicity.^19–23^ Such biases impact on the external validity of RCTs, and ‘real-world’ observational data are increasingly required to provide context and evaluate treatment effectiveness across the whole patient population.^24–27^

The Haematological Malignancy Research Network’s [www.hmrn.org] population-based patient cohort was specifically established in the UK in 2004 to address the needs outlined above by producing ‘real-time’, robust generalizable data on haematological malignancies to inform contemporary clinical practice and research: locally, nationally and internationally.^28^ With core support from Bloodwise [www.bloodwise.org.uk], formerly Leukaemia and Lymphoma Research, HMRN is the result of a unique collaboration between university researchers, National Health Service (NHS) clinicians and patients/carers.

## Who is in the cohort?

Established in September 2004, HMRN’s cohort was initiated at a time when cancer care in England was co-ordinated through a series of area-based Cancer Networks. HMRN’s catchment covers two such adjacent Cancer Networks: the Yorkshire Cancer Network and the Humber & Yorkshire Coast Cancer Network. Health geography changed in April 2013 when Cancer Networks were incorporated into Strategic Clinical Networks, but HMRN’s boundaries were not affected.

**Figure 1:**
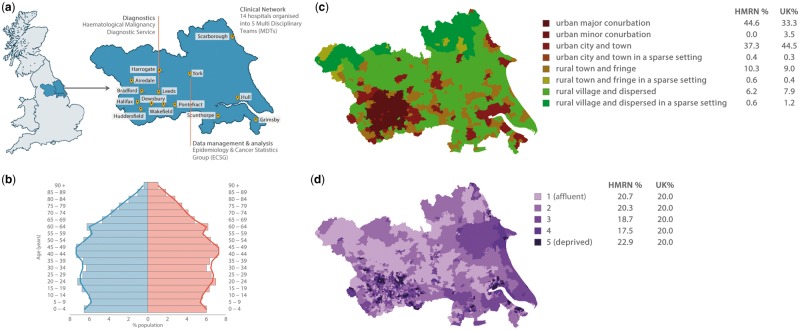
Haematological Malignancy Research Network (HMRN). **A,** Study location. **B,** Population age and sex distribution. **C,** Urban/rural distribution (Office of National Statistics definitions). **D,** Index of multiple deprivation (IMD): income domain.

Patient care across the HMRN region is provided by a unified clinical network that works to common guidelines and operates across 14 hospitals, organized into five multidisciplinary teams (MDTs) and a network-wide paediatric oncology service (Figure 1A). Importantly, with a population of around 3.8 million, the sociodemographic structure of HMRN’s study area is broadly similar to the UK as a whole (Figure 1B–D).

As a matter of policy, within HMRN all haematological cancer diagnoses (whether originating from the NHS or private sources, and irrespective of age, prognosis and treatment intent) are reported and coded using the latest WHO ICD-O classification by clinical haematopathology specialists at the Haematological Malignancy Diagnostic Service, HMDS [www.hmds.info]. Cited in the Department of Health’s Cancer Reform Strategy as the model for delivery of complex diagnostic services, HMDS houses all of the relevant technology and expertise required to diagnose and monitor haematological cancers.^29^^,^^30^

**Figure 2:**
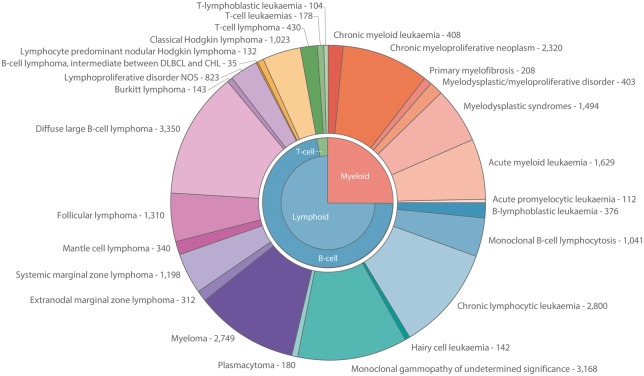
Diagnostic distribution of haematological malignancies classified by ICD-O3; HMRN 2004–15.

Since September 2004, patients resident in the area have entered HMRN’s cohort on the day that they are first diagnosed with a haematological neoplasm or precursor condition. The WHO diagnostic distribution (ICD-O3) for the 11-year period September 2004 to August 2015 (*n *= 26 423) is presented in Figure 2. The corresponding frequencies and median ages at diagnosis are presented for males and females separately for subtypes with 10 or more diagnoses in Table 1; sex-rate ratios and 5-year relative survival estimates are also shown in Table 1. More information about the classification of haematological malignancies is on the study website [https://www.hmrn.org/about/classification].

**Table 1:** Numbers, median ages, age-standardized (European 2013) rates, sex-rate ratios and 5-year relative survival (UK population); HMRN 2004–15

	Total diagnoses	Median age at diagnosis (years)	Annual age-standardized (European 2013) rate per 100 000 (95% CI)	Sex-rate ratio (male/female)	5-year relative survival
All diagnoses (International Classification of Disease for Oncology 3rd edition)	26 423	70.9	71.0 (70.7–71.3)	1.5 (1.5–1.5)	71.6 (70.9–72.4)
Total myeloid	6576	72.5	17.7 (17.6–17.9)	1.5 (1.5–1.6)	56.3 (54.7–57.9)
Acute myeloid leukaemia (9727, 9861, 9871, 9866, 9895, 9896, 9920)	1629	71.8	4.4 (4.3–4.4)	1.5 (1.5–1.6)	16.8 (14.7–19.1)
Acute promyelocytic leukaemia (9866)	112	50.0	0.3 (0.3–0.3)	1.1 (0.9–1.3)	64.3 (53.5–73.3)
Chronic myeloid leukaemia (9875)	408	59.4	1.1 (1.0–1.1)	1.5 (1.4–1.6)	89.8 (84.8–93.2)
Myelodysplastic syndromes (MDS) (9982–9986)	1494	75.8	4.1 (4.0–4.2)	2.5 (2.4–2.6)	30.2 (27.2–33.3)
Myelofibrosis (9961)	208	74.0	0.6 (0.6–0.6)	1.8 (1.6–2.0)	50.3 (40.4–59.4)
Myeloproliferative neoplasms (MPN) (9741, 9950, 9962, 9964, 9975)	2320	71.2	6.3 (6.2–6.3)	1.1 (1.0–1.1)	93.8 (91.4–95.5)
MDS/MPN (9945, 9946, 9975, 9876)	403	76.6	1.1 (1.1–1.2)	2.5 (2.3–2.7)	21.1 (15.9–26.8)
Total lymphoid	19 836	70.4	53.2 (53–53.5)	1.5 (1.5–1.5)	76.4 (75.6–77.3)
B-lymphoblastic leukaemia (9811–9816)	376	12.4	0.8 (0.8–0.9)	1.2 (1.1–1.3)	67.2 (61.7–72.1)
T-lymphoblastic leukaemia (9837)	104	17.6	0.2 (0.2–0.3)	2.1 (1.6–2.7)	64.7 (53.7–73.7)
Chronic lymphocytic leukaemia (9823)	2800	71.6	7.6 (7.5–7.7)	2.0 (2.0–2.1)	86.0 (83.7–88.0)
Hairy cell leukaemia (9940)	142	67.9	0.4 (0.4–0.4)	3.9 (3.3–4.5)	97.7 (53.9–99.9)
Myeloma (9732)	2749	73.0	7.5 (7.4–7.6)	1.7 (1.6–1.7)	48.5 (46.0–50.9)
Plasmacytoma (9731, 9734)	180	68.4	0.5 (0.5–0.5)	2.5 (2.2–2.9)	62.6 (52.7–71.0)
Extranodal marginal zone lymphoma (9699)	312	70.1	0.8 (0.8–0.9)	1.0 (0.9–1.1)	89.4 (82.8–93.6)
Systemic marginal zone lymphoma (9689)	1198	72.9	3.3 (3.2–3.3)	1.6 (1.6–1.7)	76.0 (72.1–79.4)
Follicular lymphoma (9690)	1310	65.0	3.5 (3.4–3.5)	1.0 (1.0–1.0)	87.5 (84.6–89.9)
Mantle cell lymphoma (9673)	340	74.0	0.9 (0.9–1.0)	2.5 (2.3–2.7)	43.8 (36.8–50.5)
Diffuse large B-cell lymphoma (9680)	3350	69.9	9.0 (8.9–9.1)	1.3 (1.3–1.3)	57.5 (55.5–59.4)
Burkitt lymphoma (9687)	143	53.0	0.4 (0.3–0.4)	3.8 (3.1–4.6)	51.0 (42.1–59.3)
Lymphoproliferative disorder NOS	823	77.0	2.2 (2.2–2.3)	1.6 (1.5–1.6)	81.8 (76.9–85.8)
Lymphocyte-predominant nodular Hodgkin lymphoma (9659)	132	45.0	0.3 (0.3–0.4)	3.2 (2.6–3.9)	99.2 (76.1–100)
Classical Hodgkin lymphoma (9650)	1023	41.5	2.5 (2.5–2.6)	1.3 (1.3–1.4)	85.2 (82.3–87.7)
T-cell lymphoma (9837)	430	65.7	1.1 (1.1–1.2)	1.5 (1.4–1.6)	47.1 (41.6–52.4)
T-cell leukaemias (9831–9834)	178	73.6	0.5 (0.5–0.5)	1.0 (0.9–1.1)	85.4 (74.6–91.9)
Monoclonal B-cell lymphocytosis	1041	71.9	2.8 (2.8–2.9)	1.5 (1.5–1.6)	99.4 (80.0–100)
Monoclonal gammopathy of undetermined significance (9765/1)	3168	72.9	8.7 (8.6–8.8)	1.4 (1.4–1.5)	90.5 (88.5–92.2)

NOS, not otherwise specified.

Unlike other cancers, haematological neoplasms are characterized by their ability to progress and transform; follicular lymphoma to diffuse large B-cell lymphoma, and myelodysplastic syndromes to acute myeloid leukaemia, for example.^1^^,^^2^^,^^4^ In Figure 2 and Table 1, patients are counted as the number of diagnoses they have; during the 11-year time frame, 24 859 (94.1%) patients had only one diagnosis of a haematological malignancy or precursor condition and 1564 (5.9%) patients had more than one.

## How often have they been followed up?

Patients enter the cohort when they are first diagnosed, and their molecular diagnostic/prognostic data are linked to clinical information in NHS medical records (paper and electronic) around 7 months later. Subsequently, additional linkages and abstractions are carried out, triggered either by changes in state (e.g. death, disease progression, relapse, treatment initiation) or requests for a clinical audit. All patients are ‘flagged’ at the national level for death and cancer at the Medical Research Information Service (MRIS), and routinely linked by NHS Digital to information contained within nationwide health administrative databases. Deaths are notified on a monthly basis, and linkages to cancer registrations, as well as inpatient and outpatient Hospital Episode Statistics (HES), are updated annually.

HMRN’s cohort has Section 251 support under the NHS Act 2006. Operating in much the same way as a cancer registry, this enables all patients diagnosed within the catchment to be registered and tracked through their care pathways until death, regardless of consent. Importantly, however, our procedures ensure that if at any point a patient dissents from data collection, all data relating to them held on university servers are destroyed, and linked data are no longer requested from NHS Digital.

In addition to core data collection and follow-up, a number of studies have been nested within the HMRN cohort and others are planned for the future. Some of these projects require more detailed information to be collected from clinical records (specific events surrounding diagnosis and deaths, for example), and others collect information directly from consenting individuals at various points along the patient pathway. All study leaflets and forms can be found and downloaded from the website [https://www.hmrn.org/resources/documents].

## What has been measured?

### Core data

Sociodemographic details are available for all patients, with area-based population counts and measures of deprivation being sourced from UK national data. In addition, information is obtained via linkage to routinely compiled NHS health administrative databases; this includes inpatient and outpatient hospital activity, as well as cancer registrations (preceding and succeeding the index cancer diagnosis) and death notifications.

Molecular diagnostic and prognostic data are available for all points along the patient pathway where biological samples (e.g. peripheral blood, bone marrow trephine/aspirate, lymph node, cerebrospinal fluid) are taken for the purposes of disease identification and monitoring. This biological information, which varies with diagnostic category, includes histology, immunohistochemistry, flow cytometry, fluorescence in situ hybridization, next-generation sequencing and gene expression profiling. In addition to these electronic data feeds, disease-specific templates are used to abstract additional primary source data in the clinical setting; the information collected includes individual components of staging investigations, copies of scans, performance scores and treatments (including stem cell transplants), with response and outcome being recorded for all episodes along the pathway. With a view to adhering as closely as possible to clinical trial standards in the real-world setting, these data are abstracted according to tightly controlled standard operating procedures, which include consistency checks and periodic review. The data manual, containing all form templates and instructions for data collection, is on the study website [www.hmrn.org/resources].

### Nested studies

HMRN was established with a view to providing the core infrastructure into which additional projects could be nested. Some of these projects have required more detailed information to be collected from medical records at particular points along the patient pathway. One such example is the collection of more detailed information about the routes to diagnosis of patients diagnosed with mature B-cell neoplasms, and another relates to patient management in the time leading to death. Other projects collect information directly from consenting individuals; core data are supplemented with information from various sources, including questionnaires. For example, around 4–8 weeks after diagnosis, all patients who are well enough to provide informed consent (as assessed and confirmed by a member of their clinical team) are sent a study pack about HMRN and invited to complete a survey about their symptoms before diagnosis and their current quality of life (EQ-5D-5L). Those who agree are sent further quality of life surveys at various intervals thereafter.

## What has it found? Key findings & publications

HMRN’s maturing longitudinal data provide an increasingly valuable resource with which to address real-world questions of concern to researchers, clinicians, commissioners, regulators and patients. Some of the key topics tackled since the cohort’s inception are briefly described below, and an up-to-date list of publications and reports is provided on the study’s website [https://www.hmrn.org/publications].

### Descriptive epidemiology

The production and dissemination of high quality descriptive information is a core aim of the project, and our first paper on this topic provided annual incidence estimates for 24 main disease categories^31^: population-based rates stratified by age, sex and socioeconomic status (as measured by area-based deprivation/affluence), age-standardized (European) rates, and estimated cases for the UK as a whole. The analyses revealed distinctive age and gender patterning for several myeloid and lymphoid subtypes, the male rate being two to three times higher than the female rate for several cancers, the differences being evident in both children and adults. As the cohort has grown, increasingly granular analyses have been conducted, revealing even larger descriptive differences between subtypes, as well as marked variations in overall and relative survival.^32–34^

Comparing patterns and trends is a general feature of most descriptive epidemiological reports. Importantly, although HMRN frequencies for most subtypes cannot be directly compared with national programmes (where data are coded to ICD-10, and progressions and transformations are not always recorded), cross-checks with local cancer registries have confirmed the superior quality of HMRN’s data.^35^ Furthermore, our incidence rates are in line with expectations for subtypes where comparisons can be made; our acute leukaemia and Hodgkin lymphoma rates, for example, [www.hmrn.org] are broadly similar to the most recent estimates published by SEER (Surveillance, Epidemiology and End Results) and CRUK (Cancer Research UK).^31–33^^,^^36^

With respect to broader dissemination, the descriptive section of our website has undoubtedly been one of the cohort’s most important innovations, providing information that cannot be found elsewhere [https://www.hmrn.org/statistics]. The public pages provide up-to-date information for researchers and clinicians on incidence, prevalence and relative survival; selection tools allow users to pick specific disorders, stratify by age and sex and, for measures of disease occurrence, aggregate subtypes. The diagnosis and person-based tables that underpin the website are updated annually and deaths are updated monthly. At the time of writing (October 2017), the statistics are based on 26 423 diagnoses occurring from September 2004 through August 2015, with all patients followed up to May 2017.

### Determinants of survival

HMRN’s data have reached the level of maturity required to systematically investigate and monitor the many sociodemographic, biological and treatment-related factors that impact on outcome in the general patient population, and this is a major focus of much of our current research. Thus far, with a view to gaining insight into the general nature of the relationship between age, deprivation and treatment, we have examined the topic in two cancers. Both of these are managed with standard therapy: the potentially curable aggressive lymphoma (diffuse large B-cell lymphoma, DLBCL) and the currently incurable, but potentially controllable, chronic myeloid leukaemia (CML). In the former, patient’s performance status was found to be more predictive of survival than chronological age, with fitter patients benefiting from intensive chemotherapy across all ages.^37^ Furthermore, as with multiple myeloma,^38^ although the survival of DLBCL patients who presented as an emergency was poorer than that that of patients with similar clinical characteristics who presented via other routes,^39^ no associations between survival and socioeconomic status were detected.^37^ Socioeconomic survival inequalities have, however, been observed for CML.^36^ A once rapidly fatal cancer, it was transformed in the early 2000s into a long-term condition with a steadily rising prevalence by the introduction of orally administered tyrosine kinase inhibitors (TKIs). Evidence suggests that in the UK setting of universal health care, the survival inequalities could be due to adherence issues. This contrasts with the situation in countries like the USA, where lack of financial resource for expensive drugs is the main driver of socioeconomic inequality.

### Patient pathways

HMRN’s core data, either linked to national datasets or combined with further information from nested studies (e.g. self-reported material or details about care abstracted from medical records), have enabled examination of patient experiences at various points on the pathway, both preceding and succeeding diagnosis. Two important areas, where evidence was needed to inform policy, are diagnostic delay and end-of-life care. With respect to the former, our analysis confirmed: prolonged time to diagnosis among some disease subtypes (e.g. myeloma) but not others (e.g. acute leukaemia); commonality in certain symptoms across diseases (e.g. pain and fatigue), but specificity within others (e.g. lymphadenopathy in lymphomas, bleeding and bruising in acute leukaemias); and that whereas some symptoms were frequently reported but absent from national guidance, others were included but rarely reported by patients.^40^

Our work on the latter part of the pathway developed in response to concerns about the lack of integration between haematology and specialist palliative care (SPC) services,^41^ and the greater propensity for hospital death among haematology patients.^42^ The nested studies examining these areas revealed that around half of patients had at least one SPC referral, with the likelihood of referral increasing with duration of survival and varying by subtype, being most frequent in myeloma and least in acute leukaemia.^43^ Hospital deaths were common despite subtype (indolent or aggressive), occurring most frequently in patients dying within 3 months of diagnosis.^44^ Less than half of patients took part in a discussion about their end-of-life preferences, with those who did not being significantly more likely to die in hospital. Of those who did have a discussion, a quarter stated a preference to remain in hospital at the time of their death,^45^ a much higher proportion than reported in studies including patients with other conditions.^46^ Our nested qualitative studies found that such differences are due to the close relationship between haematology staff and their patients, and that uncertain disease trajectories (i.e. characterized by sudden, unexpected deterioration and rapid death), are also important.^47^

### Health economics

The continued emergence of new approaches to diagnosis and treatment mean that haematological malignancies are among the most expensive cancers to treat, consistently coming in the top three of most economically developed countries’ cancer spend lists.^48–50^ However, in the past most of the evidence on treatment costs and health-related quality of life (HRQoL) has emanated either from single institutions or from clinical trials, which are often selective, with poor generalizability to the patient population as a whole. Hence, it is now recognized that appraisals require information about the likely impact in ‘real-world’ settings, an area in which our longitudinal data are making meaningful contributions.^51^^,^^52^

## What are the main strength and weaknesses?

HMRN’s major strengths include its large well-defined catchment area, centralized world-class diagnostics, completeness of case ascertainment, adherence to National treatment guidelines, and detailed follow-up of all patients. All of these combine to ensure that the patient cohort is not affected by the data quality issues faced by many population-based cancer registries. Predicated on infrastructures within the NHS, where universal health care is freely provided on the basis of clinical need, HMRN occupies a unique forefront position in relation to the provision of real-time data concerning the impact of diagnostic and treatment developments.

With respect to limitations, although most haematological malignancies exhibit comparatively little geographical variation, a few are regionally very specific. The most well-known examples are: adult T-cell leukaemia/lymphoma (ATLL), which develops in approximately 5% of those infected with the RNA virus HTLV-1 that is endemic to parts of Japan, South America, Papua New Guinea, Africa and the Middle East; and African endemic Burkitt lymphoma, which is largely restricted to the malarial belts of equatorial Africa, Papua New Guinea, and parts of South Amerca. Clearly HMRN data cannot be used to investigate these subtypes. Furthermore, although HMRN’s patient cohort can be used to answer many important questions, the absence of a comparison cohort of unaffected individuals impacts on investigations requiring background rates of comorbidity and/or procedures. This is, however, currently being rectified; an anonymized comparison cohort, comprising 10 age-, sex- and region of residence-matched individuals per patient, has recently been selected from primary care registers and linked to the same administrative databases as the patients (HES, cancer and death). The methods and outputs for this project will be described in a future report.

## Can I get hold of the data? Where can I find out more?

Although ethical permissions and agreements with providers of national data mean that potentially identifiable data cannot be transferred or accessed off site, HMRN data are contributing to several ongoing research projects. For information on how to collaborate with HMRN researchers and investigate questions of interest, please e-mail [enquiries@hmrn.org]. Additional contact details are provided on the website [www.hmrn.org.]

**Profile in a nutshell**

- HMRN’s ongoing population-based patient cohort was specifically established in the UK in 2004 to provide ‘real-time’, robust generalizable data on haematological malignancies to inform research and contemporary clinical practice: locally, nationally and internationally.
- All patients (∼2400 each year) newly diagnosed with a haematological malignancy or precursor condition (reported using the latest WHO ICD-O classification, currently ICD-03) in a representative UK population of around 3.8 million people are tracked through their care pathways, ‘flagged’ for death and cancer at the national Medical Research Information Service (MRIS) and linked to Hospital Episode Statistics (HES).
- HMRN operates with Section 251 support under the NHS Act 2006, enabling all patients to be followed up; procedures ensure that if a patient dissents all pseudonymized data held for research purposes are destroyed. The dataset currently contains information on around 30 000 patients (September 2017); this includes demographic variables, diagnostic and prognostic data, complete treatment pathways, markers of response and outcome, and linkage to health and administrative records.
- HMRN [www.hmrn.org] is funded by Bloodwise [www.bloodwise.org.uk] and is a collaboration between university researchers, National Health Service (NHS) clinicians, and patients/carers. Contact [enquiries@hmrn.org] to obtain more information on how to collaborate with HMRN researchers and investigate questions of interest.

## Funding

This work was supported by Bloodwise [grant number 15037], and has ethics approval (REC 04/01/1205/69) from Leeds West Research Ethics Committee, R&D approval from each NHS Trust and exemption from Section 251 of the Health & Social Care Act (PIAG 1–05(h)/2007).

**Conflict of interest:** None declared.
